# Polyethylene Glycol (PEG-400): An Efficient and Recyclable Reaction Medium for the Synthesis of Pyrazolo[3,4-*b*]pyridin-6(7*H*)-one Derivatives

**DOI:** 10.3390/molecules181113139

**Published:** 2013-10-24

**Authors:** Xiaoxing Zhong, Guolan Dou, Deming Wang

**Affiliations:** School of Safety Engineering, China University of Mining &Technology, Xuzhou 221116, Jiangsu, China

**Keywords:** pyrazolo[3,4-*b*]pyridine-6(7*H*)-one, recyclable polyethylene glycol, aqueous media, synthesis

## Abstract

A mild and efficient synthesis of pyrazolo[3,4-*b*]pyridine-6(7*H*)-one derivatives via a three-component reaction of an aldehyde, Meldrum’s acid and 3-methyl-1*H*-pyrazol-5-amine using recyclable polyethylene glycol (PEG)-400 as a reaction medium is described. This method has the advantages of accessible starting materials, good yields, mild reaction conditions and begin environmentally friendly.

## 1. Introduction

Pyrazolo[3,4-*b*]pyridine-6-ones are a promising class of heterocyclic compounds that have been shown to have potential in the treatment of several diseases, including bipolar disorder, diabetes, dementia, Alzheimer’s disease, schizophrenia, depression and cancer [1,2]. Pyrazolo[3,4-*b*]pyridine-6-ones were first synthesized by condensing ethylidenemalonic acid diethyl ester and 1-ethyl-5-amino methylpyzole in refluxing dimethylformamide and water for 94 h and resulted in a 53% yield of the target product [3]. Quiroga *et al.* [4]. reported the synthesis of dihydropyrazolo[3,4-*b*]pyridine-6-ones by refluxing equimolar amounts of aminopyrazole and the appropriate Meldrum’s acid benzylidene derivatives in nitrobenzene for 30 min, resulting in yields ranging from 42% to 72%. Martinez-Teipel *et al.* [5]. developed a new synthetic route to this class of compound in 37%–92% yield involving an intermolecular cyclization of 2-methoxy-6-oxo-1,4,5,6-tetrahydropyridine-3-carbonitriles with hydrazines. Dress *et al.* [1]. also reported the synthesis of these compounds in 30%–75% yield by using condensation reactions that involved refluxing aminopyrazole with the appropriate aldehyde and dimedone derivatives in ethanol for 6–8 h. Recently, an efficient synthsis of pyrazolo[3,4-*b*]pyridine-6-one derivatives using intermolecular cyclization reactions under solvent-free microwave conditions and ultrasound irradiation conditions was reported, resulting in yields of 40%–60% and 60%–95%, respectively [6]. Shi *et al.* [7] reported the synthesis of 3-methyl-1,4-disubstituted-4,5-dihydro-1*H*-pyrazolo[3,4-*b*]pyridine-6(7*H*)-ones *via* the three-component L-proline-catalyzed reaction of an aldehyde, 3-methyl-1-phenyl-1*H*-pyrazol-5-amine, and Meldrum's acid. However, although these methods have successfully led to a large library synthesis of pyrazolo[3,4-*b*]pyridine-6-ones, many of them still suffer from drawbacks such as requiring the use of harmful organic reagents, unsatisfactory yields and long reaction times. Therefore, a method with higher yield and environmentally-friendly manipulation needs to be developed.

Multi-component reactions (MCRs), in which multiple reactions are combined into a synthetic operation have been used extensively in synthetic chemistry to form carbon-carbon bonds [8,9,10,11,12,13,14,15]. Such reactions offer a wide range of possibilities for the efficient construction of highly complex molecules in a single procedural step, thus avoiding the complicated purification operations and allowing savings of both solvents and reagents. In the past decade, there has been tremendous development in the area of three- and four-component reactions, and great efforts continue to be made to develop new MCRs [16,17,18,19,20,21,22,23,24]. Recently, Mamaghani *et al.* reported an one-pot three-component reaction for the synthesis of novel derivatives of pyrazolo[3,4-*b*]pyridine-6(7*H*)-ones in 3–4 min with excellent yields (87%–95%) from 5-amino-3-methyl-1*H*-pyrazole, Meldrum’s acid and aryl aldehydes under ultrasonic irradiation [25]. Nevertheless, this method needs specific experimental facilities, which limits its use in mass production.

Over the years, there has been a growing recognition that water has become an attractive medium for many organic reactions, such as Diels-Alder reactions [26], Claisen rearrangement reactions [27,28], Reformatsky reactions [29,30] and pinacol-coupling reactions [31], not only for the advantages concerning the avoidance of expensive drying reactions, catalysts and solvents, but also for some unique reactivity and selectivity [32,33,34,35]. On the other hand, organic reactions in water without using harmful organic solvents is one of the current focuses today, especially in our current environmentally conscious society, because water is abundant, nontoxic and environmentally-friendly when compared with the traditionally used organic solvents. As we all know, poly(ethylene glycol) (PEG) is a thermally stable, inexpensive, recoverable, and non-toxic hydrophilic polymer. Meanwhile, the high solubility of PEGs in water and several organic solvents including alcohol and acetone [36] instead of their insolubility in less polar solvents such as hexane makes them easy to recover and high performance solvents for organic reactions [37,38,39]. Therefore, the use of an obviously benign and inexpensive solvent like water and PEG could yield significant green chemistry benefits. Herein we envisaged a simple and efficient one-pot three-component protocol for the synthesis of pyrazolo[3,4-*b*]pyridine-6(7*H*)-ones in moderate to high yields, using environmentally-friendly polyethylene glycol (PEG) as a recyclable reaction medium.

## 2. Results and Discussion

**Scheme 1:**
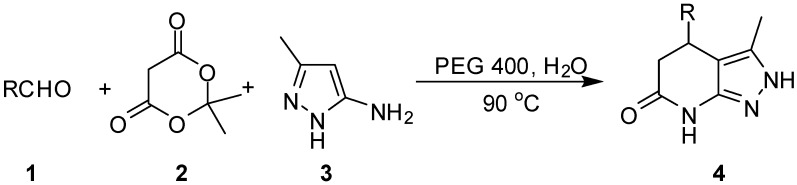
The synthesis of pyrazolo[3,4-*b*]pyridine-6(7*H*)-one derivatives in aqueous media.

When the three components, an aldehyde **1**, Meldrum’s acid (**2**) and 3-methyl-1*H*-pyrazol-5-amine (**3**) were treated in water in the presence of PEG 400 at 90 °C for about 15 min, the desired products―4-substituted-3-methyl-4,5-dihydro-2*H*-pyrazolo[3,4-*b*]pyridin-6(7*H*)-ones **4** were obtained (Scheme 1).

A range of novel valuable structures **4** were thus synthesized in good to excellent yields by this simple four-component reaction in aqueous media. The results are summarized in Table 1.

**Table 1:** The synthesis of pyrazolo[3,4-*b*]pyridine-6(7*H*)-one derivatives in aqueous media.

Entry	Product	R	Isolated Yield (%)
1	**4a**	4-CH_3_C_6_H_4_	88
2	**4b**	3-CH_3_C_6_H_4_	93
3	**4c**	4-CH_3_OC_6_H_4_	89
4	**4d**	3-CH_3_OC_6_H_4_	84
5	**4e**	4-BrC_6_H_4_	88
6	**4f**	3-BrC_6_H_4_	92
7	**4g**	2-CH_3_C_6_H_4_	89
8	**4h**	3-ClC_6_H_4_	90
9	**4i**	*n*-propyl	86

As shown in Table 1, we were pleased to find that the method was applicable to a broad substrate scope of substituted aldehydes. Aldehydes containing various electron-donating and electron-withdrawing substituents were reacted under the experimental conditions, and the corresponding products were obtained in good yields. Therefore, no remarkable electronic effects were observed in the reaction. Good yields was also obtained when the alkyl alhedyde butyraldehyde was reacted (Table 1, entry 9).

With regard to sustainable chemistry issues, reagent recyclability is an important question. The separation of the products and the reaction medium were explored for the synthesis of product **4a** in PEG-400-H_2_O. We were pleased to find that the entire reaction medium could be successfully recycled for up to five runs with limited loss of activity (the yield decreased from 93% to 70% after 5 runs, Table 2).

**Table 2:** Recycling and reuse of PEG-400-H_2_O.

Run	Yield (%)
1	93
2	89
3	82
4	76
5	70

**Figure 1:**
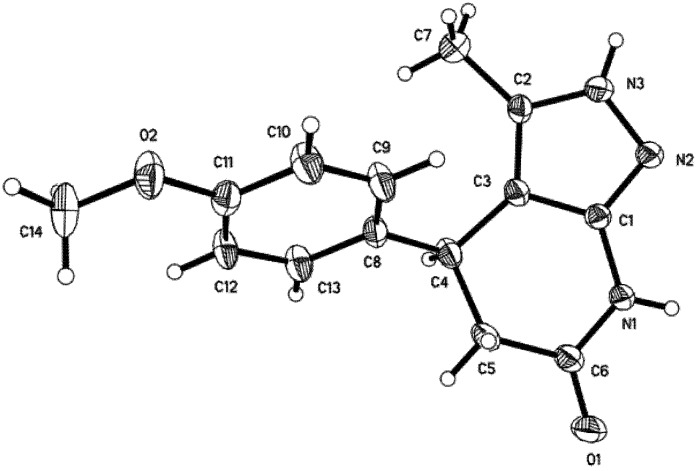
Molecular structure of **4c**.

All the products were characterized by ^1^H-NMR, IR and HRMS spectra. The structure of compound **4c** was further confirmed by X-ray diffraction analysis [40]. The molecular structure of **4c** is shown in Figure 1.

**Scheme 2:**
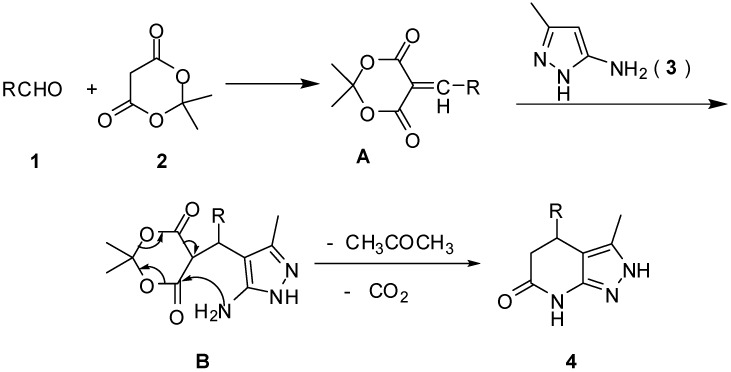
Possible mechanism for the formation of product **4**.

Although the mechanism of the reaction has not yet been established, a possible explanation is proposed in Scheme 2. The reaction might thus proceed via sequential condensation, addition, cyclization, and elimination. First, a Knoevenagel condensation between aldehydes **1** with Meldrum’s acid (**2**) affords intermediate **A**. The Michael addition of **A** with 3-methyl-1*H*-pyrazol-5-amine (**3**) would then furnish the intermediate product **B**, which subsequently undergoes an intramolecular cyclization and then releases acetone and carbon dioxide to give product **4**.

## 3. Experimental

### 3.1. General Information

Commercial solvents and reagents were used as received. IR spectra were obtained on a Nicolet 6700 spectrophotometer. ^1^H-NMR spectra were recorded using a Bruker DPX-400 MHz instrument, at 293 K unless otherwise noted, with the residual peaks of the solvent DMSO-*d*_6_ (*δ* = 2.50) used for reference. HRMS were obtained on a micromass GCT-TOF instrument. X-Ray crystallographic analysis was performed with a Rigaku Mercury diffractometer.

### 3.2. General Procedure for the Synthesis of Pyrazolo[3,4-b]pyridine-6(7H)-ones **4** in Aqueous Media

To a stirred solution of polyethylene glycol (PEG)-400 (5 mL) in water (10 mL), aldehyde **1** (2 mmol), Meldrum’s acid (**2**, 2 mmol) and 3-methyl-1*H*-pyrazol-5-amine (**3**, 2 mmol) were added and the mixture stirred at 90 °C, until the reaction was complete as indicated by TLC (about 15 min). After completion of the reaction, the crystalline powder formed was collected by filtration, washed with water and recrystallized from ethanol to give pure **4**. The recovered PEG with water was reused for further cycles.

*3-Methyl-4-p-tolyl-4,5-dihydro-2H-pyrazolo[3,4-[3,4-b]pyridin-6(7H)-one* (**4a**). M.p. >300 °C; IR (KBr): 3203, 3160 cm^−1^ (NH), 1650 cm^−1^ (C=O). ^1^H-NMR (DMSO-*d*_6_) *δ*: 1.87 (s, 3H, CH_3_), 2.26 (s, 3H, CH_3_), 2.54 (d, *J* = 6.0 Hz, 1H, CH), 2.74–2.78 (m, 1H, CH), 4.09 (t, *J* = 6.4 Hz, 1H, CH), 7.04–7.12 (m, 4H, ArH), 10.27 (s, 1H, NH), 11.79 (s, 1H, NH); HRMS: *m/z* calcd. for C_14_H_16_N_3_O: 242.12879 (M+H); found 242.12880.

*3-Methyl-4-m-tolyl-4,5-dihydro-2H-pyrazolo[3,4-b]pyridin-6(7H)-one* (**4b**). M.p. >300 °C; IR (KBr): 3200, 3150 cm^−1^ (NH), 1640 cm^−1^ (C=O). ^1^H-NMR (DMSO-*d*_6_) *δ*: 1.83 (s, 3H, CH_3_), 2.26 (s, 3H, CH_3_), 2.54 (d, *J* = 6.0 Hz, 1H, CH), 2.74–2.79 (m, 1H, CH), 4.09 (t, *J* = 6.4 Hz, 1H, CH), 7.04–7.12 (m, 4H, ArH), 10.27 (s, 1H, NH), 11.79 (s, 1H, NH); HRMS: *m/z* calcd. for C_14_H_16_N_3_O: 242.12879 (M+H); found 242.12878.

*4-(4-Methoxyphenyl)-3-methyl-4,5-dihydro-2H-pyrazolo[3,4-b]pyridin-6(7H)-one* (**4c**). M.p. >300 °C; IR (KBr): 3180, 3155 cm^−1^ (NH), 1640 cm^−1^ (C=O). ^1^H-NMR (DMSO-*d*_6_) *δ*: 1.83 (s, 3H, CH_3_), 2.72–2.78 (m, 2H, CH_2_), 3.73 (s, 3H, OCH_3_), 4.09 (t, *J* = 6.4 Hz, 1H, CH), 6.86–6.88 (m, 2H, ArH), 7.07–7.09 (m, 2H, ArH),10.25 (s, 1H, NH), 11.78 (s, 1H, NH); HRMS: *m/z* calcd. for C_14_H_16_N_3_O_2_: 258.12370 (M+H); found 258.12369.

*4-(3-Methoxyphenyl)-3-methyl-4,5-dihydro-2H-pyrazolo[3,4-b]pyridin-6(7H)-one* (**4d**). M.p. >300 °C; IR (KBr): 3180, 3150 cm^−1^ (NH), 1640 cm^−1^ (C=O). ^1^H-NMR (DMSO-*d*_6_) *δ*: 1.85 (s, 3H, CH_3_), 2.56 (d, *J* = 5.6 Hz, 1H, CH), 2.78–2.84 (m, 1H, CH), 3.72 (s, 3H, OCH_3_), 4.17 (t, *J* = 6.4 Hz, 1H, CH), 6.73–6.81 (m, 3H, ArH), 7.23 (t, *J* = 8.0 Hz, 1H, ArH), 10.28 (s, 1H, NH), 11.81 (s, 1H, NH); HRMS: *m/z* calcd. for C_14_H_16_N_3_O_2_: 258.12370 (M+H); found 258.12372.

*4-(4-Bromophenyl)-3-methyl-4,5-dihydro-2H-pyrazolo[3,4-b]pyridin-6(7H)-one* (**4e**). M.p. >300 °C; IR (KBr): 3190, 3160 cm^−1^ (NH), 1643 cm^−1^ (C=O). ^1^H-NMR (DMSO-*d*_6_) *δ*: 1.85 (s, 3H, CH_3_), 2.56 (d, *J* = 5.6 Hz, 1H, CH), 2.78–2.84 (m, 1H, CH), 4.17 (t, *J* = 6.4 Hz, 1H, CH), 7.13–7.15 (m, 2H, ArH), 7.50–7.52 (m, 2H, ArH), 10.32 (s, 1H, NH), 11.85 (s, 1H, NH); HRMS: *m/z* calcd. for C_13_H_13_BrN_3_O: 306.02365 (M+H); found 306.02362.

*4-(3-Bromophenyl)-3-methyl-4,5-dihydro-2H-pyrazolo[3,4-b]pyridin-6(7H)-one* (**4f**). M.p. >300 °C; IR (KBr): 3190, 3160 cm^−1^ (NH), 1640 cm^−1^ (C=O). ^1^H-NMR (DMSO-*d*_6_) *δ*: 1.86 (s, 3H, CH_3_), 2.50–2.58 (m, 1H, CH), 2.79–2.83 (m, 1H, CH), 4.17 (t, *J* = 6.0 Hz, 1H, CH), 7.16–7.41 (m, 4H, ArH), 10.33 (s, 1H, NH), 11.86 (s, 1H, NH); HRMS: *m/z* calcd. for C_13_H_13_BrN_3_O: 306.02365 (M+H); found 306.02371.

*3-Methyl-4-o-tolyl-4,5-dihydro-2H-pyrazolo[3,4-b]pyridin-6(7H)-one* (**4g**). M.p. >300 °C; IR (KBr): 3180, 3150 cm^−1^ (NH), 1645 cm^−1^ (C=O). ^1^H-NMR (DMSO-*d*_6_) *δ*: 1.73 (s, 3H, CH_3_), 2.37 (s, 3H, CH_3_), 2.39–2.47 (m, 1H, CH), 2.73–2.78 (m, 1H, CH), 4.34 (t, *J* = 6.4 Hz, 1H, CH), 6.88–7.20 (m, 4H, ArH), 10.30 (s, 1H, NH), 11.81 (s, 1H, NH); HRMS: *m/z* calcd. for C_14_H_16_N_3_O: 242.12879 (M+H); found 242.12871.

*4-(3-Chlorophenyl)-3-methyl-4,5-dihydro-2H-pyrazolo[3,4-b]pyridin-6(7H)-one* (**4h**) M.p. >300 °C; IR (KBr): 3190, 3155 cm^−1^ (NH), 1645 cm^−1^ (C=O). ^1^H-NMR (DMSO-*d*_6_) *δ*: 1.86 (s, 3H, CH_3_), 2.77 (dd, *J*_1_ = 5.6 Hz, *J*_2_ = 16 Hz, 1H, CH), 2.79–2.84 (m, 1H, CH), 4.20 (t, *J* = 6.0 Hz, 1H, CH), 7.14–7.35 (m, 4H, ArH), 10.34 (s, 1H, NH), 11.87 (s, 1H, NH); HRMS: *m/z* calcd. for C_13_H_13_ClN_3_O: 262.07417 (M+H); found 262.07416.

*3-methyl-4-propyl-4,5-dihydro-2H-pyrazolo[3,4-b]pyridin-6(7H)-one* (***4i***) M.p. 259–260 °C; IR (KBr): 3175, 3140 cm^−1^ (NH), 1645 cm^−1^ (C=O). ^1^H-NMR (DMSO-*d*_6_) *δ*: 0.85–0.86 (m, 3H, CH_3_), 1.17–1.93 (m, 4H, 2CH_2_), 2.18 (s, 3H, CH_3_), 2.24–2.26 (m, 1H, CH), 2.77–2.78 (m, 1H, CH), 3.51 (t, *J* = 7.2 Hz, 1H, CH), 10.13 (s, 1H, NH), 11.66 (s, 1H, NH); HRMS: *m/z* calcd. for C_10_H_16_N_3_O: 194.12879 (M+H); found 194.12874.

## 4. Conclusions

In summary, we have demonstrated a mild and highly efficient protocol for the synthesis of 4-substituted-3-methyl-4,5-dihydro-2*H*-pyrazolo[3,4-*b*]pyridin-6(7*H*)-ones in excellent yields by using recyclable polyethylene glycol (PEG)-400 as a reaction medium. Environmental acceptablility, high yields, easy work-up, cleaner reaction profiles, environmentally friendly solvent, and recyclability of PEG are the notable features of this protocol.
